# Supplementary material for: A chemogenetic approach for dopamine imaging with tunable sensitivity
Source: Nat Commun. 2024 Jul 2;15:5551. doi: 10.1038/s41467-024-49442-3 (PMC11219860; doi:10.1038/s41467-024-49442-3)
Supplement: Supplementary file 1 — Supplementary Information [file 41467_2024_49442_MOESM1_ESM.pdf]

Supplementary Information

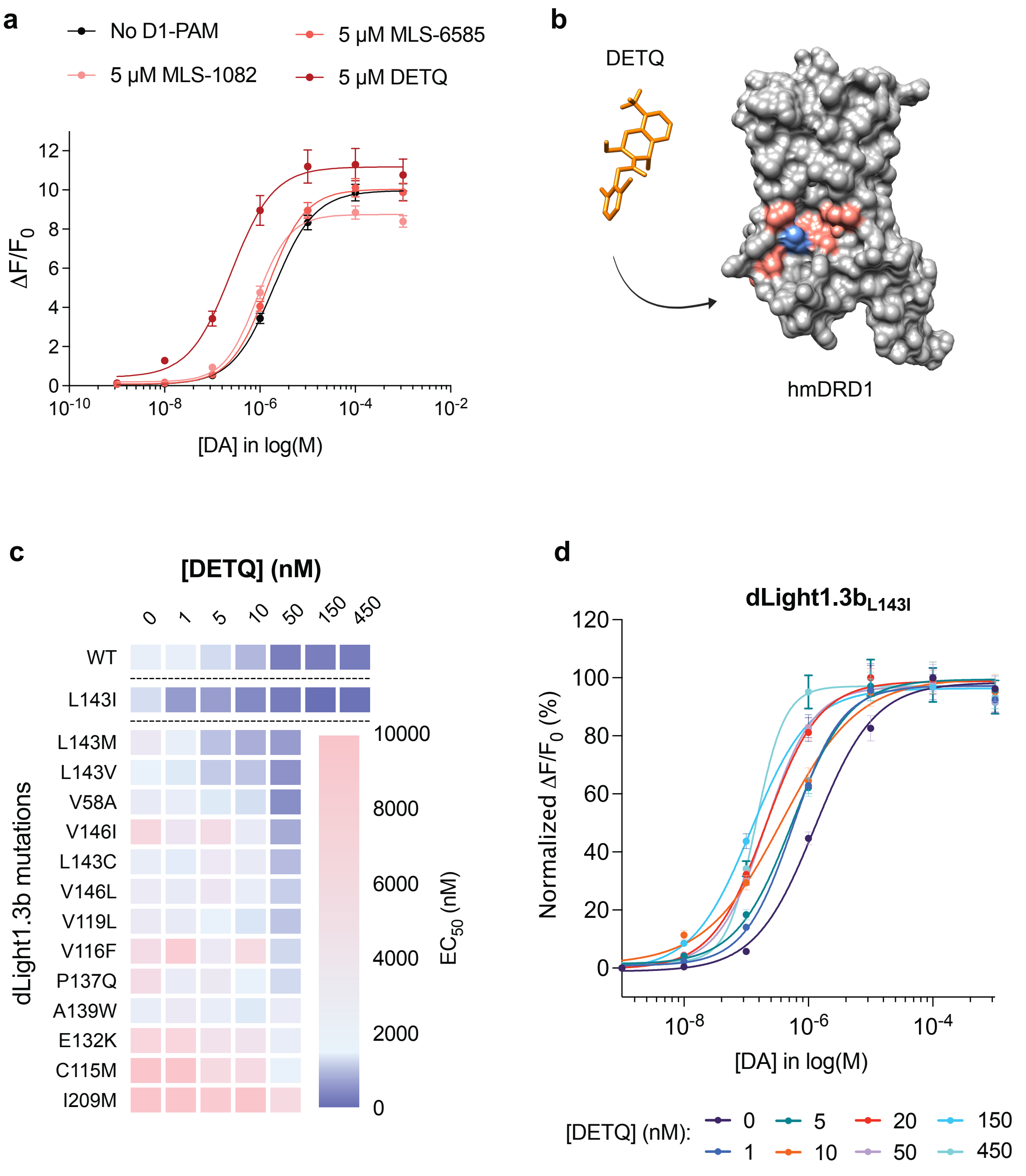

Supplementary Figure 1 | Comparison of various D1-PAMs and engineering of dLight$_{L143I}$

a, Dose-response curves obtained from DA titrations on dLight1.3b-expressing HEK293 cells in the presence or absence of indicated D1-PAMs (5 μM). Data are shown as fluorescence fold-change over baseline ($\Delta F/F_0$). Datapoints were fit using a log(agonist) vs. response non-linear fit (four parameters) to determine EC_{50} values. n=14-21 cells from 3 independent experiments for each titration. **b**, In silico generated structure of DETQ replacing the LY3154207 compound in the D1-PAM binding pocket of the human DRD1 (PDB:7LJD). Aminoacid sites on the intracellular loop-2 of human DRD1 that were mutagenized during screening are highlighted in pink or in blue for residue L143. **c**, Screening results for the 14 dLight1.3b variants tested to identify dLight$_{L143I}$, a mutant with increase DETQ affinity. Colored tiles represent average EC_{50} values from DA dose-responses for each variant in the presence of different concentrations of DETQ. EC_{50} values are color-coded from low (high-affinity, blue) to high (low-affinity, pink), as shown in the heatmap on the right. **d**, Dose-response curves obtained from DA titrations on dLight$_{L143I}$-expressing HEK293 cells in the presence or absence of DETQ (100 nM). n=16-30 cells from 3 independent experiments for each titration. All data are shown as mean ± SEM.

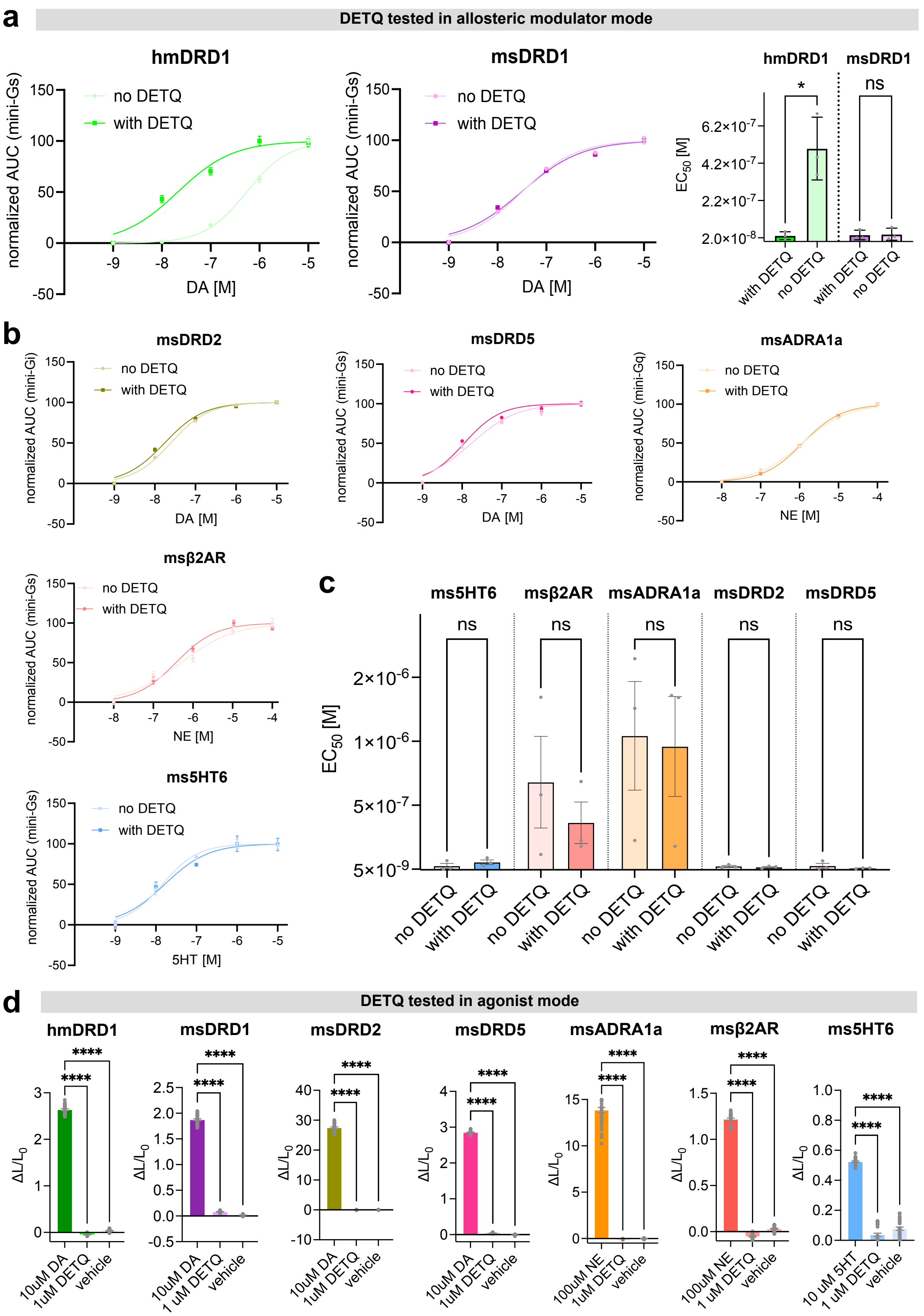

a
DETQ tested in allosteric modulator mode
hmDRD1
msDRD1
no DETQ
with DETQ
normalized AUC (mini-Gs)
DA [M]
EC50 [M]
*
ns
b
msDRD2
msDRD5
msADRA1a
msβ2AR
ms5HT6
normalized AUC (mini-Gi)
normalized AUC (mini-Gq)
NE [M]
5HT [M]
c
d
DETQ tested in agonist mode
ΔL/L0
****
10uM DA
1uM DETQ
1 uM DETQ
vehicle
100uM NE
10 uM 5HT

Supplementary Figure 2 | Characterization of GPCR target selectivity of DETQ

a, Left, luminescence-based DA dose-response curves of mini-Gs recruitment to hmDRD1 (left) and msDRD1 (right) performed in HEK293T cells in presence or absence of 100 nM DETQ. Values were normalized to maximal luminescence for DA of each receptor and condition. Datapoints were fitted with four-parameter dose-response curves to determine EC_{50} values. Right, statistical comparison of DA EC_{50} values from curves shown on left in the presence or absence of DETQ. n = 3 independent wells for each receptor. Two-tailed Students t-test with Welch's correction; P = 0.0156 (hmDRD1), P = 0.8857 (msDRD1). **b,** same as in **a** for msDRD2, msDRD5, msADRA1a msβ2AR and ms5HT6 in absence or presence of 1 uM DETQ during dose-responses of agonist ligands: DA, NE or 5HT, as indicated. n = 3 independent wells for each receptor. **c,** Statistical comparison of agonist ligands EC_{50} values in presence or absence of DETQ for individual GPCRs, as indicated. P = 0.4671 (msDRD2), P = 0.4272 (msDRD5), P = 0.9993 (msADRA1a), P = 0.4831 (msβ2AR), P = 0.3482 (ms5HT6). **d,** Statistical comparison of $\Delta L/L_0$ of indicated receptors to their agonist ligand (1 uM), DETQ (1 uM) or vehicle, each applied alone in separate wells. Statistical analysis was performed using Welch's ANOVA followed by Brown-Forsyth test. ****P-values: hmDRD1, $P = 2.297 \times 10^{-46}$ for DETQ, $P = 6.269 \times 10^{-50}$ for vehicle; msDRD1, $P = 3.607 \times 10^{-29}$ for DETQ, $P = 1.38 \times 10^{-40}$ for vehicle; msDRD2, $P = 1.958 \times 10^{-38}$ for DETQ, $P = 1.951 \times 10^{-38}$ for vehicle; msDRD5, $P = 1.817 \times 10^{-57}$ for DETQ, $P = 1.588\ 10^{-59}$ for vehicle; msADRA1a, $P = 2.85 \times 10^{-29}$ for DETQ, $P = 2.957 \times 10^{-29}$ for vehicle; msβ2AR, $P = 5.921 \times 10^{-46}$ for DETQ, $P = 1.411 \times 10^{-47}$ for vehicle; ms5HT6, $P = 4.528 \times 10^{-33}$ for DETQ, $P = 8.316 \times 10^{-32}$ for vehicle. All data are shown as mean ± SEM.

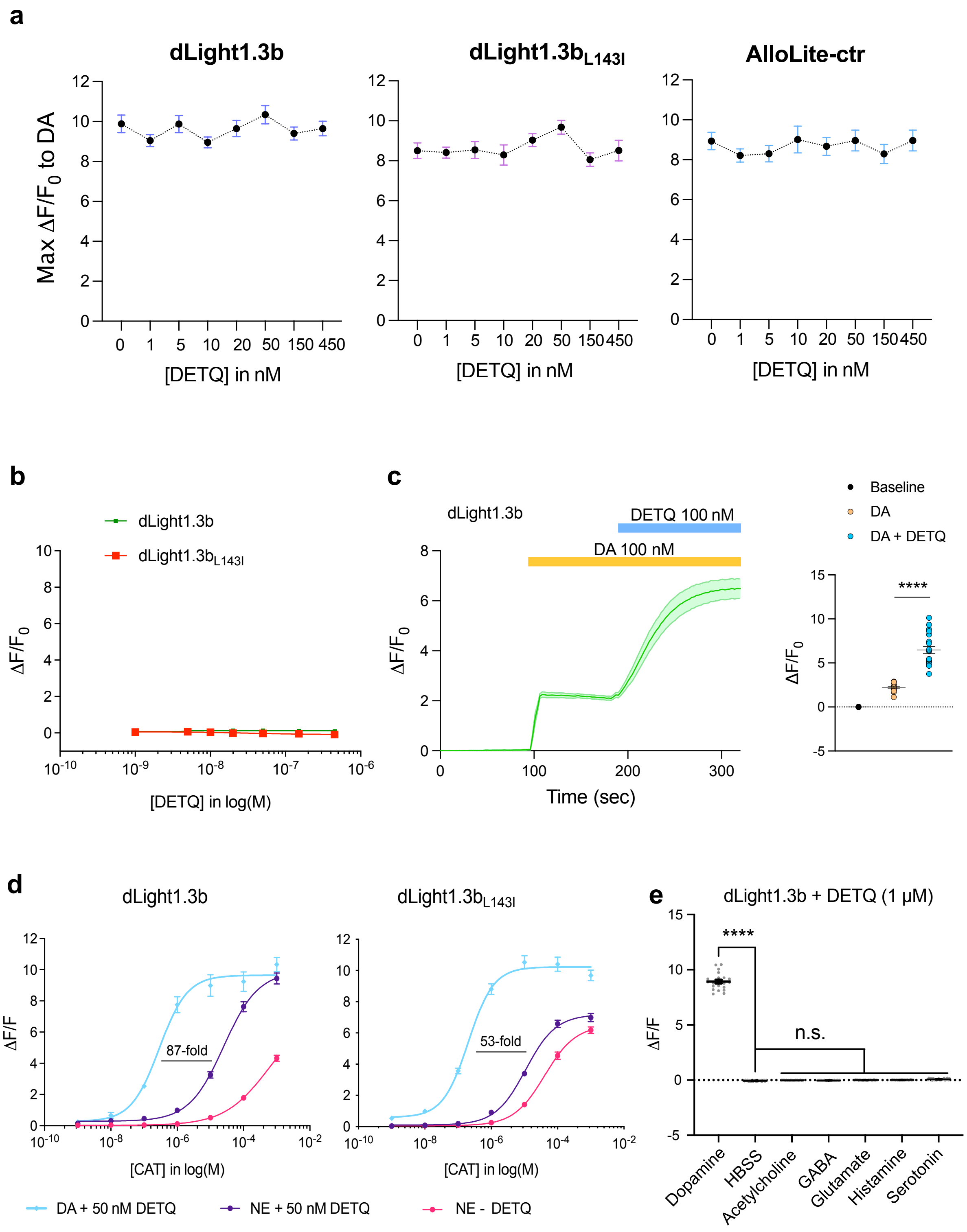

a
dLight1.3b
dLight1.3bL143I
AlloLite-ctr
Max ΔF/F0 to DA
[DETQ] in nM
b
dLight1.3b
dLight1.3bL143I
ΔF/F0
[DETQ] in log(M)
c
dLight1.3b
DETQ 100 nM
DA 100 nM
Time (sec)
Baseline
DA
DA + DETQ
****
d
dLight1.3b
dLight1.3bL143I
87-fold
53-fold
ΔF/F
[CAT] in log(M)
DA + 50 nM DETQ
NE + 50 nM DETQ
NE - DETQ
e
dLight1.3b + DETQ (1 μM)
****
n.s.
Dopamine
HBSS
Acetylcholine
GABA
Glutamate
Histamine
Serotonin

Supplementary Figure 3 | Characterization of DETQ effects on dLight1.3b-derived sensors

a, Comparison of fluorescence responses of different sensors (dLight1.3b, dLightL_{143I}, AlloLite-ctr) to 1 mM DA (Max $\Delta F/F_0$) in the presence of increasing DETQ concentrations. n=10-63 cells from 3 independent experiments for each dataset. **b**, DA sensor fluorescent responses in the absence of DA upon exposure to increasing DETQ concentrations. n=20-28 cells from 3 independent experiments. **c**, Left, fluorescent responses of dLight1.3b to subsequent applications of 100 nM DA and 100 nM DETQ on the same cells. Note the clear potentiatory effect of DETQ on the response of dLight1.3b to a subsaturating concentration of DA. Right, quantification of maximal response from left. n=20 cells from 3 independent experiments. ****p<0.0001. Two-tailed Student's t-test. **d**, Dose-response curves obtained from DA or norepinephrine (catecholamines, CAT) titrations on dLight1.3b or dLight$_{L143I}$-expressing HEK293 cells in the presence or absence of DETQ (50 nM), as indicated. Data are shown as fluorescence fold-change over baseline ($\Delta F/F_0$). Datapoints were fit using a log(agonist) vs. response non-linear fit (four parameters) to determine EC_{50} values. n=9-63 cells from 3 independent experiments for each titration. **e**, Fluorescence response of dLight1.3b in the presence of 1 µM DETQ in HEK293T after application of different neurotransmitters (10 µM). Welch ANOVA with Dunett's multiple comparison test. n of cells for each neurotransmitter: 21. P values: DA, P = 9.751^{-29}; Ach, P = 4.875^{-05}; GABA, P = 0.002671; Glu, P = 3.03^{-07}; Hist, P = 2.16^{-08}; Ser, P = 3.95^{-09}. All data are shown as mean ± SEM.

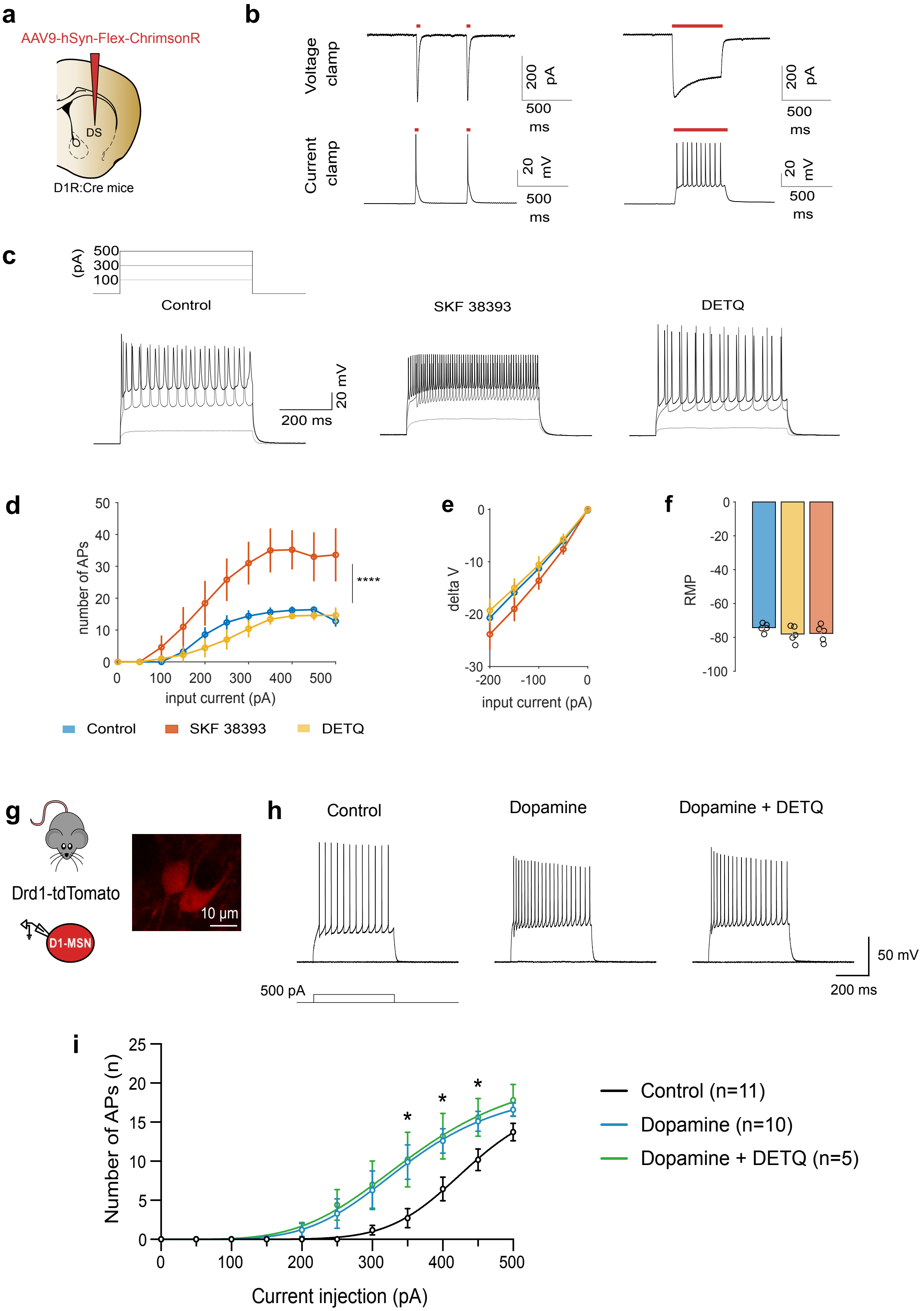

a
AAV9-hSyn-Flex-ChrimsonR
DS
D1R:Cre mice
b
Voltage clamp
Current clamp
200 pA
500 ms
20 mV
500 ms
c
(pA)
500
300
100
Control
SKF 38393
DETQ
20 mV
200 ms
d
number of APs
input current (pA)
****
e
delta V
input current (pA)
f
RMP
Control
SKF 38393
DETQ
g
Drd1-tdTomato
10 µm
D1-MSN
h
Control
Dopamine
Dopamine + DETQ
500 pA
50 mV
200 ms
i
Number of APs (n)
Current injection (pA)
Control (n=11)
Dopamine (n=10)
Dopamine + DETQ (n=5)

Supplementary Figure 4 | Electrophysiological characterization of DETQ effects on D1-MSNs
a, Schematic representation of experimental procedures for slice electrophysiology. A cre-dependent ChrimsonR AAV was injected in the dorsal striatum (DS) of DRD1:cre mice for optogenetic identification of D1-MSNs. **b**, Optogenetic identification of D1-MSNs. Brief pulses of red light (630 nm LED) were used to excite ChrimsonR+ cells. D1-MSNs were identified by the detection of light-induced action potentials and membrane depolarization in current or voltage-clamp mode, respectively. **c**, Example voltage traces from optogenetically-identified D1-MSNs recorded in the absence of any drugs (control), or in the presence of DETQ (200 nM) or SKF38393 (10 μM) in the ACSF perfusion. **d**, Number of action potentials (APs) evoked by current injections in D1-MSNs in the presence or absence of DETQ or SKF38393. n=8, 7, 6 neurons for control, DETQ, SKF38393, respectively. ****$p<0.0001$, two-way ANOVA with Tukey's multiple comparisons test. **e**, Input resistance of D1-MSNs in the presence or absence of DETQ or SKF38393. n=5 neurons. **f**, Resting membrane potential of D1-MSNs in the presence or absence of DETQ or SKF38393. n=5 neurons. **g**, Experimental schematic and example 2P image in acute brain slice showing tdTomato fluorescence in D1-MSNs. **h**, Example traces showing action potential firing evoked by 500 pA current injection in D1-MSNs, in the presence of DA (10 μM) with or without DETQ (200 nM). **i**, Summary of number of action potentials (APs) evoked by current ejection (0-500 pA, 500 ms) in the presence of DA with or without DETQ. * $p<0.05$ control vs. DA, Fisher's LSD. Note that no significant difference was found between DA with or without DETQ at any current injection. All data are shown as mean ± SEM.

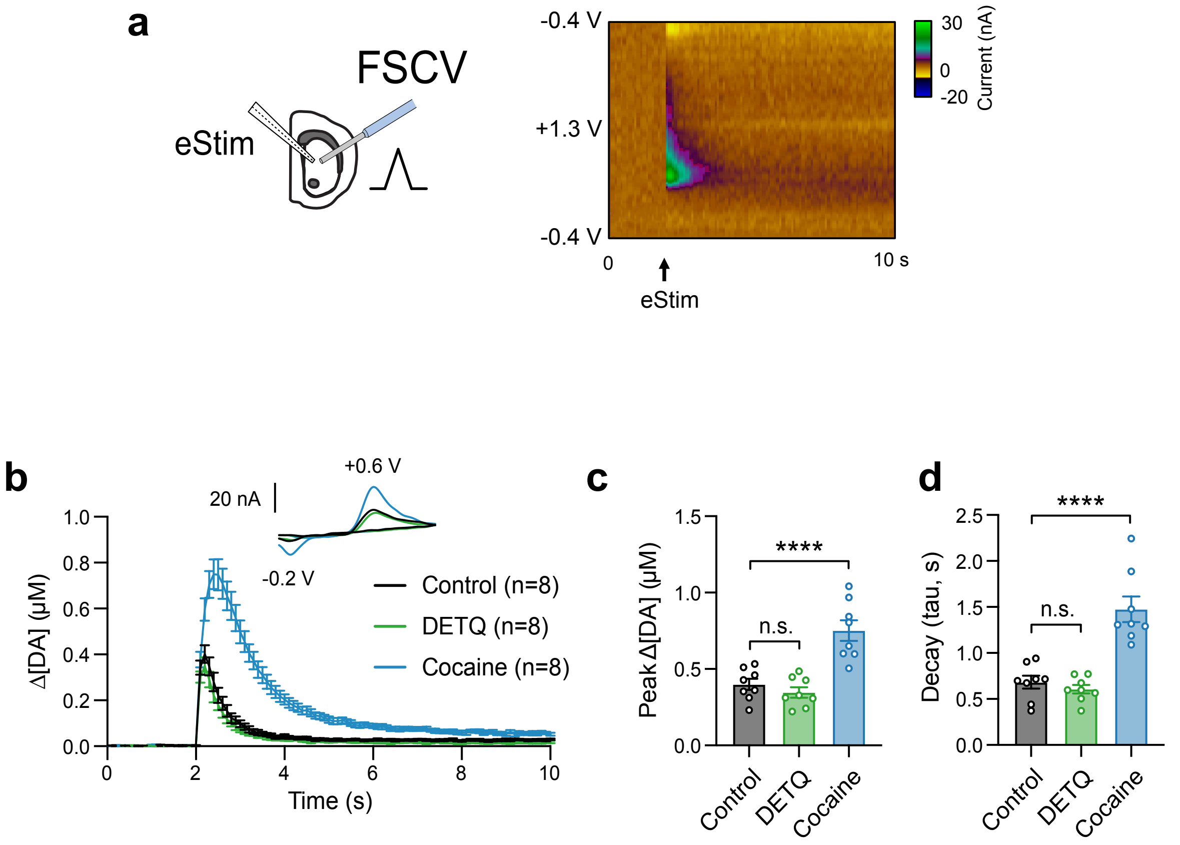

Supplementary Figure 5 | Lack of DETQ effect on the endogenous DA transporter
a, Experimental schematic and example color plot showing background-subtracted non-faradaic current evoked by electrically stimulated (single pulse, 30 μA, 0.5 ms) DA release. **b**, Time-course of electrically stimulated DA release in the presence of DETQ (200 nM) or cocaine (5 μM). Inset: Background-subtracted voltammograms in the presence of DETQ or cocaine showing oxidation peak at +0.6 V and reduction peak at -0.2 V, characteristic of DA. **c**, Summary of peak DA release in DETQ and cocaine. **** $p<0.0001$, $t(21)=5.12$, Sidak's multiple comparisons test. **d**, Decay of DA release, measured as the time constant (tau) of single exponential fit, in the presence of DETQ or cocaine. **** $p<0.0001$, $t(21)=5.97$, Dunnett's multiple comparisons test.

a

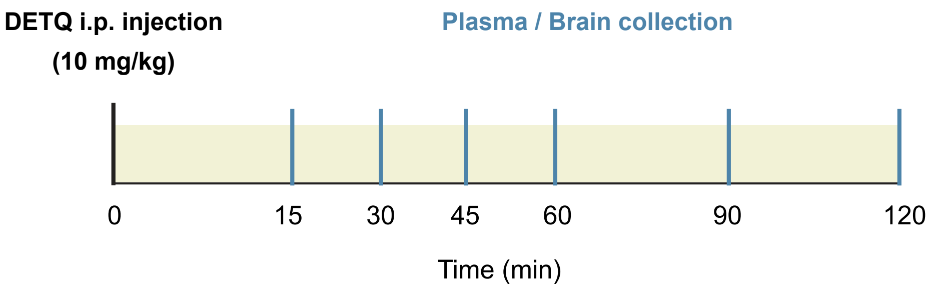

b

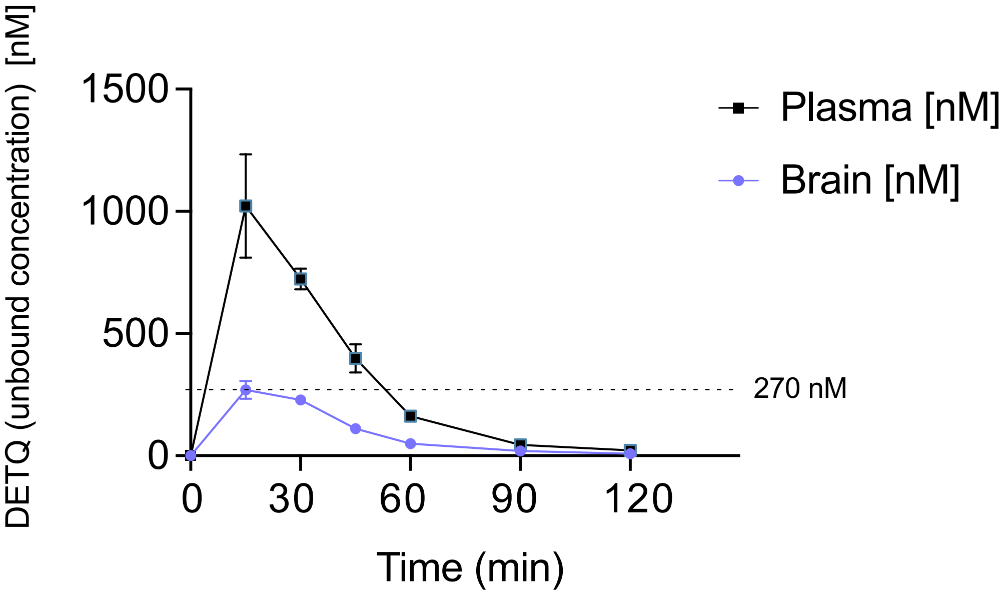

Supplementary Figure 6 | Pharmacokinetics of DETQ in brain and plasma
a, Schematic timeline of pharmacokinetic study. Samples of blood plasma and brain were collected at the timepoints indicated by blue lines. **b**, Concentrations (nanomolar) of unbound (not albumin-bound) DETQ in blood plasma and brain CSF at the timepoints indicated. Dashed line indicates 270 nM concentration of unbound DETQ. n=4 mice/timepoint. All data are shown as mean ± SEM.

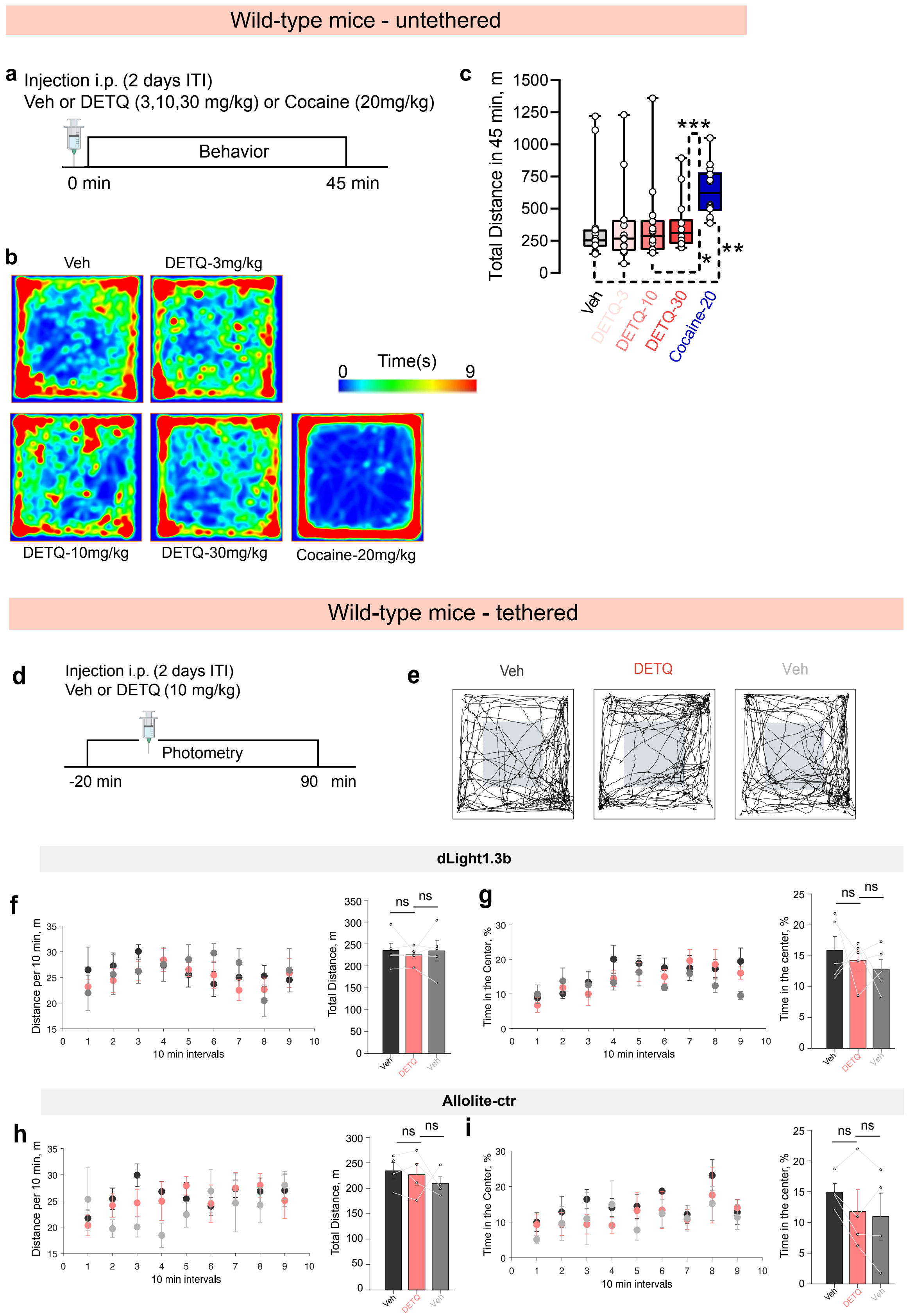
Wild-type mice - untethered
a
Injection i.p. (2 days ITI)
Veh or DETQ (3,10,30 mg/kg) or Cocaine (20mg/kg)
Behavior
0 min
45 min
b
Veh
DETQ-3mg/kg
Time(s)
0
9
DETQ-10mg/kg
DETQ-30mg/kg
Cocaine-20mg/kg
c
Total Distance in 45 min, m
Veh
DETQ-3
DETQ-10
DETQ-30
Cocaine-20
Wild-type mice - tethered
d
Injection i.p. (2 days ITI)
Veh or DETQ (10 mg/kg)
Photometry
-20 min
90 min
e
Veh
DETQ
Veh
dLight1.3b
f
Distance per 10 min, m
10 min intervals
Total Distance, m
ns
ns
g
Time in the Center, %
10 min intervals
Time in the center, %
Allolite-ctr
h
Distance per 10 min, m
10 min intervals
Total Distance, m
i
Time in the Center, %
10 min intervals
Time in the center, %

Supplementary Figure 7 | Control experiments showing lack of DETQ effects on mouse behavior
a, Timeline for behavioral analysis in untethered mice: mice were injected i.p. with vehicle, DETQ (3, 10 or 30 mg/kg) or cocaine (20mg/kg) and tested immediately in an open field for 45 min. **b,** Mouse location during open field testing. Color code (blue to red) denotes time spent (short to long) at a given location. **c,** Locomotion across a 45 min open field. Cocaine, *$p<0.05$, **$p<0.01$, ***$p<0.001$; DETQ, all $p>0.999$). n=14 mice/group (all mice were tested on all DETQ doses), one-way repeated-measures ANOVA, main effect of drug: $p < 0.0001$; Holm-Sidak post-hoc: Veh vs. Cocaine: $p=0.0067$; DETQ-3 vs. Cocaine: $p = 0.0036$; DETQ-10 vs. Cocaine: $p=0.0119$; DETQ-30 vs. Cocaine: $p=0.0006$; Veh vs. DETQ-3 or DETQ-10 or DETQ-30: $p=0.9998$. Whiskers denote the min, max and median. **d,** Same as **a** in tethered mice. **e,** Locomotion traces across 3 days with Veh day1, DETQ, Veh day 2 injections respectively. **f,** Locomotor activity and total distance moved during the 90 minutes of the open field (OF) session ($p=0.74$ for Veh on day 1 vs. DETQ injections, $p=0.78$ for DETQ vs Veh on day 2 injection, , $p>0.99$ for Veh on day1 vs. 2 injection; n=5 mice). **g,** Time spent in the center of OF in 10 minutes intervals (left) and total time in center ($p=0.81$ for Veh on day 1 vs. DETQ injections, $p = 0.84$ for DETQ vs Veh on day 2 injection, $p=0.50$ for Veh on day1 vs. 2 injection; n=5 mice). **h-i,** same as **f-g** but for mice injected with AlloLite-ctr. Total distance moved: $p=0.95$ for Veh on day 1 vs. DETQ injections, $p=0.78$ for DETQ vs Veh on day 2 injection, $p=0.62$ for Veh on day1 vs. 2 injection; n=4 mice. Total time in center: $p=0.42$ for Veh on day 1 vs. DETQ injections, $p=0.93$ for DETQ vs Veh on day 2 injection, $p=0.27$ for Veh on day1 vs. 2 injection; n=4 mice. One-way ANOVA by Tukey post-hoc test for multiple-group comparisons. Graphics in **a,d** were created in Biorender.com. All data are shown as mean ± SEM.

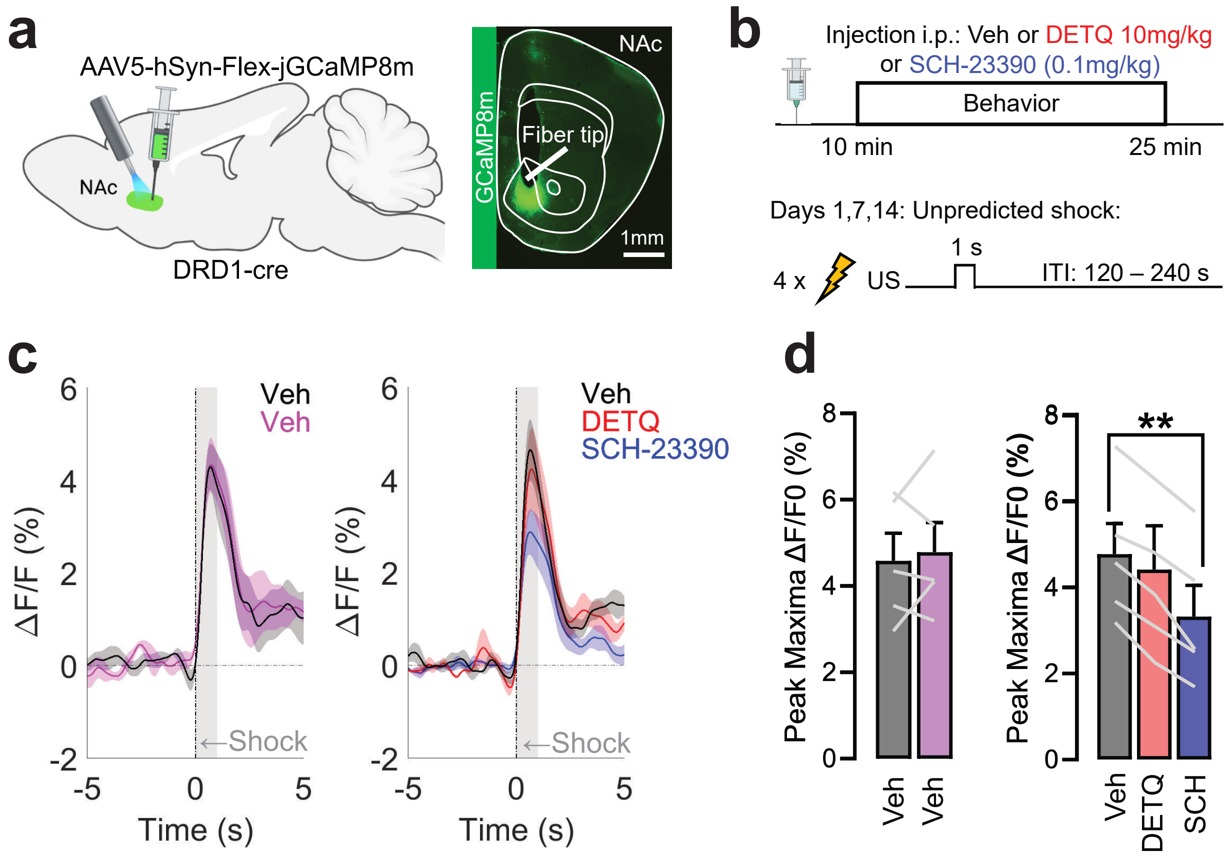

Supplementary Figure 8 | Lack of DETQ effects on D1-MSN activity in an aversive task. a. Virus strategy and representative anatomy image: DRD1-cre mice were injected with an AAV expressing the calcium indicator jGCaMP8m in the NAc. Optic fibers were implanted above the NAc to record jGCaMP8m fluorescence. **b.** Top: Experimental timeline, showing mice were injected i.p. with vehicle on Day 1 and with either Veh or with DETQ (10 mg/kg) on Day 7. A subgroup was injected with the DRD1-antagonist SCH-23390 (0.1mg/kg) on Day 14. Injections occurred 10 min before the first shock. Bottom: Unpredicted footshock protocol. Graphics in **a,b** were created in Biorender.com. **c.** Average jGCaMP8m ΔF/F0 (%) fluorescence response showing increased activity of DRD1-MSN at the onset of unpredicted shock. Grey shaded area indicates the duration of the unpredicted shock. **d.** Quantification of shock-evoked $\Delta F/F_0$ response (peak maxima in the 0-10 sec window post-shock) shows that injection of the DRD1 positive allosteric modulator DETQ does not increase the shock-induced response, while the DRD1 antagonist SCH-23390 (0.1mg/kg), as expected, reduces it (**$p<0.01$). n=5 mice Veh/Veh: t-test, $t(4) = 0.4965$, $p = 0.6456$. n=5 mice Veh/DETQ: 1-way ANOVA, $F(1.349, 5.398) = 13.44$, $p = 0.0104$, Sidak post hoc test: VEH vs. DETQ: $t(4) = 1.109$, $p = 0.6987$; VEH vs SCH: $t(4) = 8.796$, $p = 0.0028$; DETQ vs SCH: $t(4) = 3.103$, $p = 0.1045$.

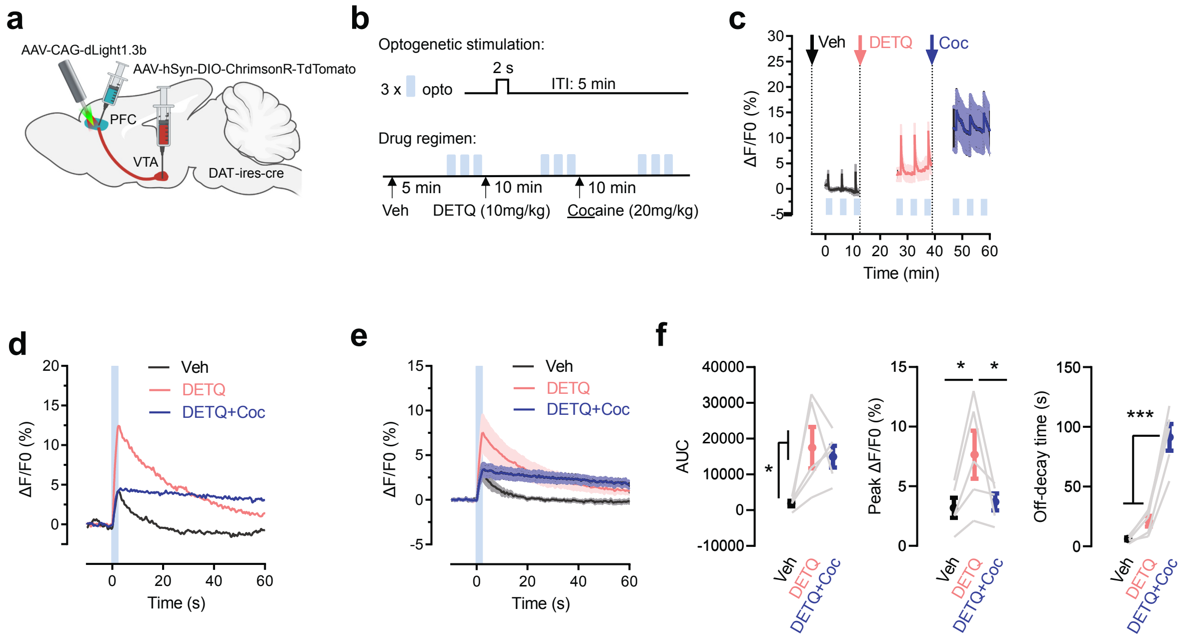

Supplementary Figure 9 | dLight responses to DETQ in PFC are sensitive to cocaine. a. Virus strategy: DAT-ires-cre mice were injected with a cre-dependent AAV expressing the opsin ChrimsonR in the VTA, and an AAV expressing the DA sensor dLight1.3b in the PFC. Optic fibers were implanted above the PFC. Brain schematics were created in Biorender.com. **b**. Experimental timeline: mice were injected i.p. with vehicle, DETQ (10 mg/kg) and the DA reuptake inhibitor cocaine (20 mg/kg) at 15-20 min intervals. 10 min after each injection, DA axons were optically (opto) activated across 3 trials (2s, 20Hz, 20-ms pulses) every 5 min. **c**. Average traces showing opto-evoked trials (blue lines) over time. A clear increase in baseline fluorescence upon administration of cocaine is visible. **d**. Representative opto-evoked trace aligned to opto onset. **e**. Average opto-evoked traces aligned to opto onset. **f**. Quantification of opto-evoked trials shows a significant increase in AUC with DETQ or DETQ+cocaine vs. vehicle (*$p<0.05$), a significant increase in peak ΔF/F0 with DETQ vs. other groups (*$p<0.05$) and a significant increase in peak ΔF/F0 off-decay time with DETQ+cocaine vs. other groups (***$p<0.001$). Peak ΔF/F0: n = 5/group, 1-way ANOVA, main effect of drug: p = 0.0219; Holm-Sidak post-hoc. Veh vs. DETQ: p = 0.0307; Veh vs. DETQ+Cocaine: p = 0.0464; DETQ vs. DETQ+Cocaine: p = 0.6033. Peak maxima, n = 5/group. 1-way ANOVA, main effect of drug: p = 0.0222; Holm-Sidak post-hoc: Veh vs. DETQ: p = 0.0345; Veh vs. DETQ+Cocaine: p = 0.7273; DETQ vs. DETQ+Cocaine: p = 0.0398. Off-decay, n = 5/group. 1-way ANOVA, main effect of drug: p < 0.0001; Holm-Sidak post-hoc: Veh vs. DETQ: p = 0.0955; Veh vs. DETQ+Cocaine: p < 0.0001; DETQ vs. DETQ+Cocaine: p < 0.0001. Data is shown as mean ±SEM.

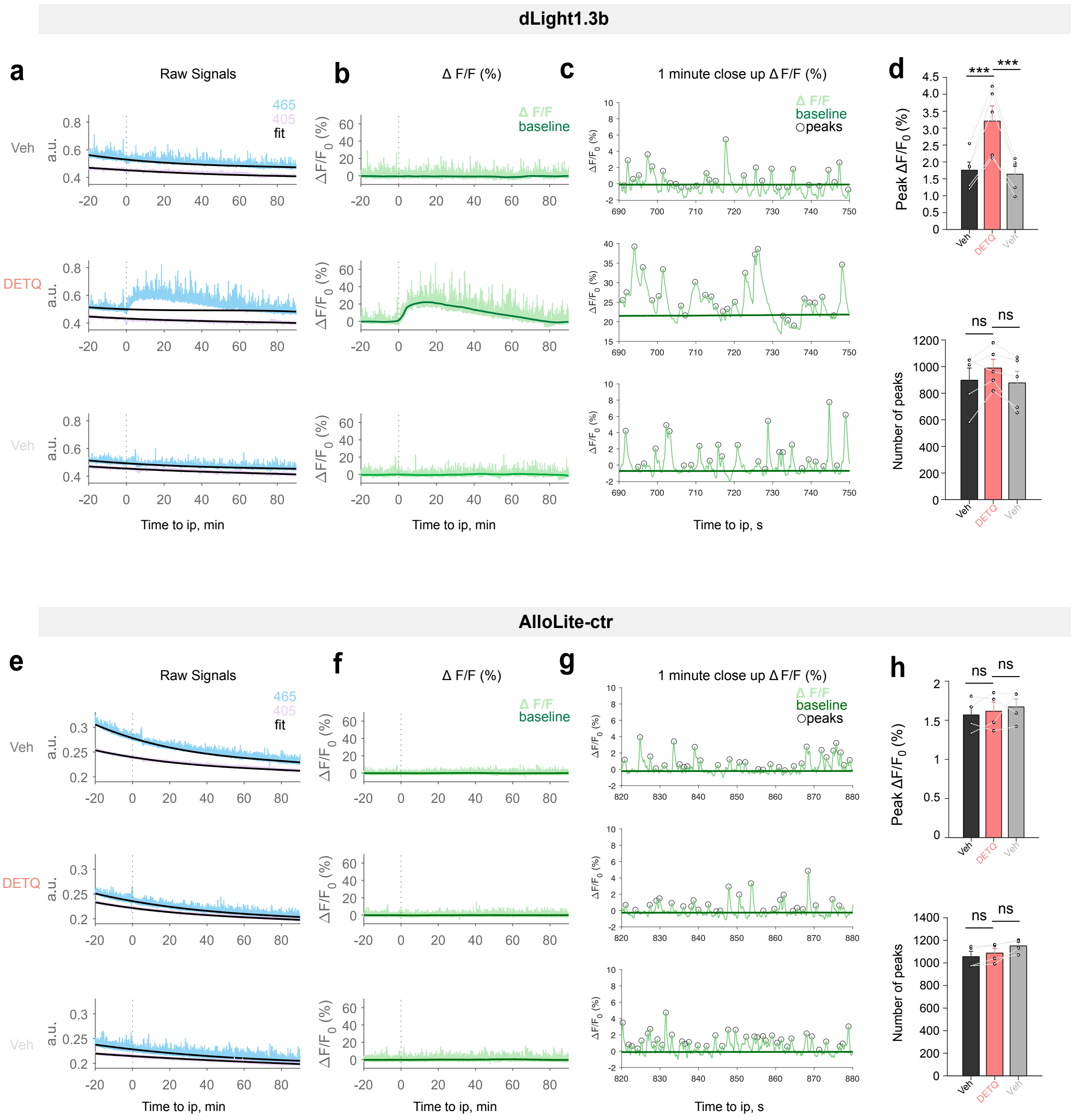
dLight1.3b
a
Raw Signals
465
405
fit
Veh
DETQ
Veh
a.u.
Time to ip, min
b
Δ F/F (%)
Δ F/F
baseline
ΔF/F0 (%)
Time to ip, min
c
1 minute close up Δ F/F (%)
Δ F/F
baseline
peaks
Time to ip, s
d
Peak ΔF/F0 (%)
***
***
Number of peaks
ns
ns
Veh
DETQ
Veh
AlloLite-ctr
e
Raw Signals
465
405
fit
Veh
DETQ
Veh
a.u.
Time to ip, min
f
Δ F/F (%)
Δ F/F
baseline
ΔF/F0 (%)
Time to ip, min
g
1 minute close up Δ F/F (%)
Δ F/F
baseline
peaks
Time to ip, s
h
Peak ΔF/F0 (%)
ns
ns
Number of peaks
ns
ns
Veh
DETQ
Veh

Supplementary Figure 10 | Representative photometry traces and processing of the data for dLight1.3b and AlloLite-ctr in the NAc

a, Example raw fiber photometry data excited at 465 nm (blue) and 405 nm (violet) during continuous recording in OF. Black lines are individual polynomial fits. **b,** $\Delta F/F_0$ (light green) is the difference between bleaching-corrected, normalized signals excited at 405 and 465 nm. **c,** A 60 seconds close - up section of the recording shown in (b). Detected peaks are black circles; individual peak amplitude was calculated as the local peak's prominence. Top row of (a), (b) and (c) show signals recorded in the session when the mouse received Veh on day 1 (20 minutes post-recording onset); middle, DETQ session; bottom, Veh day 2 session. **d,** top: on average, amplitude of detected peaks (defined as the local prominence) is ~2 fold higher in the session with DETQ (Veh day 1: 1.75 ± 0.24 %; DETQ: 3.20 ± 0.44 %; Veh on day 2: 1.63 ± 0.22 %; ***p=3.0 $\times 10^{-4}$ for Veh on day 1 vs. DETQ injections, ***p=1.7$\times 10^{-4}$, for DETQ vs Veh on day 2 injection, p=0.84 for Veh on day 1 vs day 2 injections); bottom: no significant difference was found in number of peaks detected during 5-40 minutes after ip (p = 0.18 for Veh on day 1 vs. DETQ injections, p = 0.09 for DETQ vs Veh on day 2 injection, p=0.90 for Veh on day1 vs. 2 injection). **e – g,** Same as **a-d** but with AlloLite-ctr sensor. **e,** top: no significant difference was found in amplitude of detected peaks (p = 0.91 for Veh on day 1 vs. DETQ injections, p=0.88 for DETQ vs Veh on day 2 injection, p=0.67 for Veh on day1 vs. 2 injection); bottom: no significant difference in peak number 5-40 minutes after DETQ injection (p=0.37 for Veh on day 1 vs. DETQ, p=0.05 for DETQ vs Veh on day 2 injection, *p=0.011 for Veh on day1 vs. 2 injection). One-way ANOVA and Tukey post hoc tests for multiple-group comparisons were used. All data are shown as mean ± SEM.

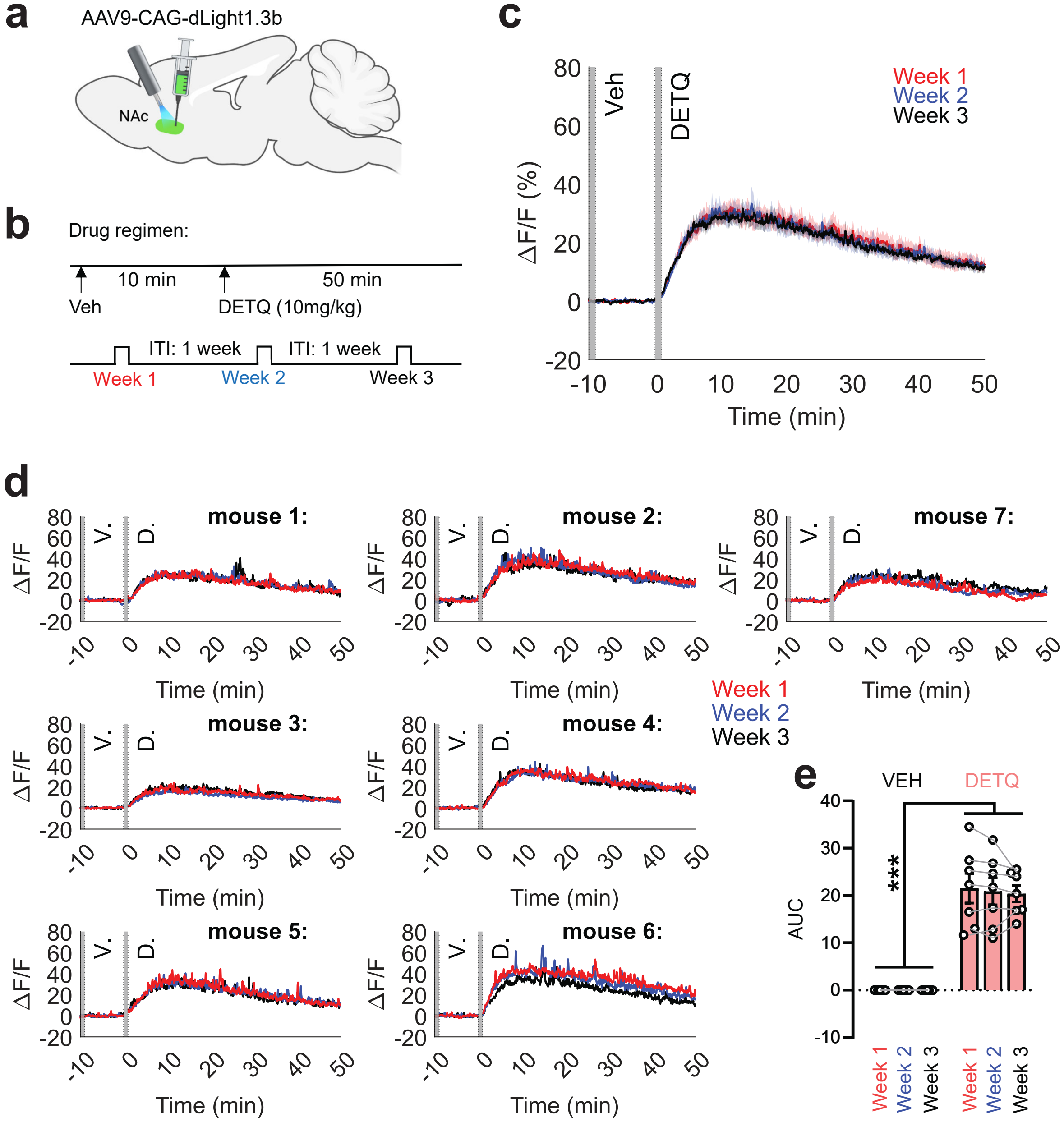
a
AAV9-CAG-dLight1.3b
NAc
b
Drug regimen:
10 min
50 min
Veh
DETQ (10mg/kg)
ITI: 1 week
ITI: 1 week
Week 1
Week 2
Week 3
c
Veh
DETQ
Week 1
Week 2
Week 3
ΔF/F (%)
Time (min)
d
mouse 1:
mouse 2:
mouse 7:
mouse 3:
mouse 4:
mouse 5:
mouse 6:
V.
D.
ΔF/F
Time (min)
Week 1
Week 2
Week 3
e
VEH
DETQ
AUC
***
Week 1
Week 2
Week 3

Supplementary Figure 11 | Inter-animal and within-animal variability in responses to DETQ. a. Virus strategy: Mice were injected with an AAV expressing the DA sensor dLight1.3b in the NAc. Optic fibers were implanted above the NAc to record dLight fluorescence. Brain schematics were created in Biorender.com. **b.** Experimental timeline: mice were injected i.p. with vehicle (epoch 1), then Veh or DETQ (10 mg/kg) 10 min later (epoch 2). The experiment was repeated 3 times, weekly **c.** Average ΔF/F0 (%) traces showing dLight recordings across all animals. Veh and DETQ injections are indicated with grey bars. **d.** ΔF/F0 (%) traces of individual mice showing dLight recordings across all animals. Veh (V.) and DETQ (D.) injections are indicated with grey bars. **e.** Quantification of the step-like increase in baseline fluorescence after DETQ shows a significant increase in AUC in the DETQ vs. vehicle condition with non negligible inter-mouse variability but minimal within-mouse variability. n = 7 mice, 1-way ANOVA, main effect of drug: $F (1.000, 6.000) = 68.10$, $p=0.0002$. AUC was calculated in the relevant drug epoch (Veh, DETQ) and normalized against the duration of that epoch (Veh: 10 min, DETQ: 50 min).

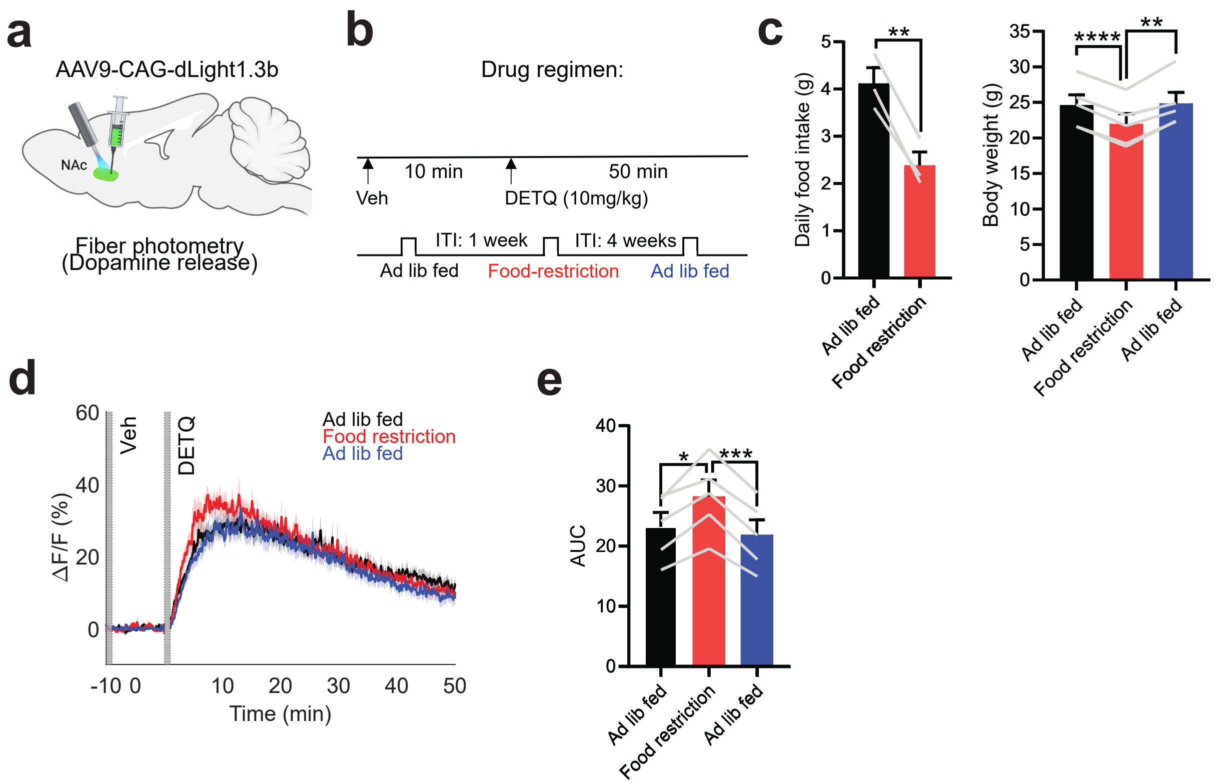

Supplementary Figure 12 | Food deprivation increases tonic DA levels, as revealed with DETQ. a. Virus strategy: DAT-ires-cre mice were injected with an AAV expressing the DA sensor dLight1.3b in the NAc. Optic fibers were implanted above the NAc to record dLight fluorescence. Brain schematics were created in Biorender.com. **b.** Experimental timeline: mice were injected i.p. with vehicle (epoch 1), then Veh or DETQ (10 mg/kg) 10 min later (epoch 2). The experiment was repeated 3 times: Week 1: ad libitum fed, Week 2: food-restricted for 1 week to 90% of baseline body weight (BW), Week 3: ad libitum; after 4 weeks of recovery from food-restriction. **c.** Food restriction leads to a significant decrease in food intake and body weight (**$p<0.01$, ****$p<0.0001$). Food intake: n=3 cages. t-test: $t(2) = 10.73$, $p=0.0086$. Body weight: n = 5 mice. 1-way ANOVA, main effect of week: $F(1.083, 4.330) = 37.44$, $p=0.0026$; Sidak post-hoc tests: food restriction vs. ad lib fed 1: $t(4) = 24.70$, $p=0.0001$; food restriction vs. ad lib fed 1: $t(4) = 6.636$, $p=0.0053$. **d.** Average ΔF/F0 (%) traces showing dLight recordings across all animals, indicating a step-like increase in dLight fluorescence upon DETQ injection, which is enhanced in the food-restriction condition, indicating elevated tonic DA levels. Veh and DETQ injections are indicated with grey bars. **e.** Quantification of the step-like increase in baseline fluorescence after DETQ indicates a significant increase in the AUC in the period immediately following DETQ injection (0-15min) (*$p<0.05$, **$p<0.001$). n = 5 mice. 1-way ANOVA, main effect of week: $F(1.368, 5.472) = 41.33$, $p=0.0007$; Sidak post-hoc tests: food restriction vs. ad lib fed 1: $t(4) = 5.269$, $p=0.0124$; food restriction vs. ad lib fed 1: $t(4) = 11.45$, $p=0.0007$.

Supplementary Note 1

DNA sequence of AlloLite-ctr

atgaggactctgaacacctctgccatggacgggactgggctggtggtggagagggacttctctgttcgtatcctcactgcctgtt
tcctgtcgctgctcatcctgtccacgctcctggggaacacgctggtctgtgctgccgttatcaggttccgacacctgcggtcca
aggtgaccaacttctttgtcatctccttggctgtgtcagatctcttggtggccgtcctggtcatgccctggaaggcagtggctgag
attgctggcttctggccctttgggtccttctgtaacatctgggtggcctttgacatcatgtgctccactgcatccatcctcaacct
ctgtgtgatcagcgtggacaggtattgggctatctccagccctgcccaatatgagagaaagatgacccccaaggcagccttc
atcgctatcagtgtggcatggaccttgtctgtactcatctccttcatcccagtgcagctcagctggcacaaggcaaaacccac
aagcccctctgatggaaatgccacttccctggctgagaccatagacaactgtgactccagcctcagcaggacatatgccat
ctcatcctctgtaatcagcttttacatccctgtggccatcatgattgtcacctacaccaggatctacaggattgctcagaaaca
gctgagctcactcattaacgtctatatcaaggccgacaagcagaagaacggcatcaaggcgaacttcaagatccgccaca
acatcgaggacggcggcgtgcagctcgcctaccactaccagcagaacacccccatcggcgacggccccgtgctgctgcc
cgacaaccactacctgagcgtgcagtccaaactttcgaaagaccccaacgagaagcgcgatcacatggtcctgctggagt
tcgtgaccgccgccgggatcactctcggcatggacgagctgtacaagggcggtaccggagggagcatggtgagcaagggc
gaggagctgttcaccggggtggtgcccatcctggtcgagctggacggcgacgtaaacggccacaagttcagcgtgtccggc
gagggtgagggcgatgccacctacggcaagctgaccctgaagttcatctgcaccaccggcaagctgcccgtgccctggcc
caccctcgtgaccaccctgacctacggcgtgcagtgcttcagccgctaccccgaccacatgaagcagcacgacttcttca
agtccgccatgcccgaaggctacatccaggagcgcaccatcttcttcaaggacgacggcaactacaagacccgcgccga
ggtgaagttcgagggcgacaccctggtgaaccgcatcgagctgaagggcatcgacttcaaggaggacggcaacatcctgg
ggcacaagctggagtacaacaatcatgaccaactgaaaagagaaactaaagtcctgaagactctgtcggtgatcatgggtg
tgtttgtgtgctgttggctacctttcttcatcttgaactgcattttgcccttctgtgggtctggggagacgcagcccttctgcattgat
tccaacacctttgacgtgtttgtgtggtttgggtgggctaattcatccttgaaccccatcatttatgcctttaatgctgattttcgga
aggcattttcaaccctcttaggatgctacagactttgccctgcgacgaataatgccatagagacggtgagtatcaataacaat
ggggccgcgatgttttccagccatcatgagccacgaggctccatctccaaggagtgcaatctggtttacctgatcccacatgc
tgtgggctcctctgaggacctgaaaaaggaggaggcagctggcatcgccagacccttggagaagctgtccccagccctatc
ggtcatattggactatgacactgacgtctctctggagaagatccaacccatcacacaaaacggtcagcacccaacc

Protein sequence of AlloLite-ctr

MRTLNTSAMDGTGLVVERDFSVRILTACFLSLLILSTLLGNTLVCAAVIRFRHLRSKVTNFFVISLAVS
DLLVAVLVMPWKAVAEIAGFWPFGSFCNIWVAFDIMCSTASILNLCVISVDRYWAISSPAQYERKM
TPKAAFIAISVAWTLSVLISFIPVQLSWHKAKPTSPSDGNATSLAETIDNCDSSLSRTYAISSSVISFYIP
VAIMIVTYTRIYRIAQKQLSSLINVYIKADKQKNGIKANFKIRHNIEDGGVQLAYHYQQNTPIGDGPVL
LPDNHYLSVQSKLSKDPNEKRDHMVLLEFVTAAGITLGMDELYKGGTGGSMVSKGEELFTGVVPIL
VELDGDVNGHKFSVSGEGEGDATYGKLTLKFICTTGKLPVPWPTLVTTLTYGVQCFSRYPDHMKQ
HDFFKSAMPEGYIQERTIFFKDDGNYKTRAEVKFEGDTLVNRIELKGIDFKEDGNILGHKLEYNNHD
QLKRETKVLKTLSVIMGVFVCCWLPFFILNCILPFCGSGETQPFCIDSNTFDVFVWFGWANSSLNPI
IYAFNADFRKAFSTLLGCYRLCPATNNAIETVSINNNGAAMFSSHHEPRGSISKECNLVYLIPHAVG
SSEDLKKEEAAGIARPLEKLSPALSVILDYDTDVSLEKIQPITQNGQHPT*

DNA sequence of dLight$_{L143I}$

atgaggactctgaacacctctgccatggacgggactgggctggtggtggagagggacttctctgttcgtatcctcactgcctgtt
tcctgtcgctgctcatcctgtccacgctcctggggaacacgctggtctgtgctgccgttatcaggttccgacacctgcggtcca
aggtgaccaacttctttgtcatctccttggctgtgtcagatctcttggtggccgtcctggtcatgccctggaaggcagtggctgag
attgctggcttctggccctttgggtccttctgtaacatctgggtggcctttgacatcatgtgctccactgcatccatcctcaacct
ctgtgtgatcagcgtggacaggtattgggctatctccagccctgcccggtatgagagaaagatgacccccaaggcagccttc
atcataatcagtgtggcatggaccttgtctgtactcatctccttcatcccagtgcagctcagctggcacaaggcaaaaccca
caagcccctctgatggaaatgccacttccctggctgagaccatagacaactgtgactccagcctcagcaggacatatgcca
tctcatcctctgtaatcagcttttacatccctgtggccatcatgattgtcacctacaccaggatctacaggattgctcagaaac
agctgagctcactcattaacgtctatatcaaggccgacaagcagaagaacggcatcaaggcgaacttcaagatccgccac
aacatcgaggacggcggcgtgcagctcgcctaccactaccagcagaacacccccatcggcgacggccccgtgctgctgc
ccgacaaccactacctgagcgtgcagtccaaactttcgaaagaccccaacgagaagcgcgatcacatggtcctgctggag
ttcgtgaccgccgccgggatcactctcggcatggacgagctgtacaagggcggtaccggagggagcatggtgagcaagggc
gaggagctgttcaccggggtggtgcccatcctggtcgagctggacggcgacgtaaacggccacaagttcagcgtgtccggc
gagggtgagggcgatgccacctacggcaagctgaccctgaagttcatctgcaccaccggcaagctgcccgtgccctggcc
caccctcgtgaccaccctgacctacggcgtgcagtgcttcagccgctaccccgaccacatgaagcagcacgacttcttca
agtccgccatgcccgaaggctacatccaggagcgcaccatcttcttcaaggacgacggcaactacaagacccgcgccga
ggtgaagttcgagggcgacaccctggtgaaccgcatcgagctgaagggcatcgacttcaaggaggacggcaacatcctgg
ggcacaagctggagtacaacaatcatgaccaactgaaaagagaaactaaagtcctgaagactctgtcggtgatcatgggtg
tgtttgtgtgctgttggctacctttcttcatcttgaactgcattttgcccttctgtgggtctggggagacgcagcccttctgcattgat
tccaacacctttgacgtgtttgtgtggtttgggtgggctaattcatccttgaaccccatcatttatgcctttaatgctgattttcgga
aggcattttcaaccctcttaggatgctacagactttgccctgcgacgaataatgccatagagacggtgagtatcaataacaat
ggggccgcgatgttttccagccatcatgagccacgaggctccatctccaaggagtgcaatctggtttacctgatcccacatgc
tgtgggctcctctgaggacctgaaaaaggaggaggcagctggcatcgccagacccttggagaagctgtccccagccctatc
ggtcatattggactatgacactgacgtctctctggagaagatccaacccatcacacaaaacggtcagcacccaacc

Protein sequence of dLight$_{L143I}$

MRTLNTSAMDGTGLVVERDFSVRILTACFLSLLILSTLLGNTLVCAAVIRFRHLRSKVTNFFVISLAVS
DLLVAVLVMPWKAVAEIAGFWPFGSFCNIWVAFDIMCSTASILNLCVISVDRYWAISSPARYERKM
TPKAAFIIISVAWTLSVLISFIPVQLSWHKAKPTSPSDGNATSLAETIDNCDSSLSRTYAISSSVISFYIP
VAIMIVTYTRIYRIAQKQLSSLINVYIKADKQKNGIKANFKIRHNIEDGGVQLAYHYQQNTPIGDGPVL
LPDNHYLSVQSKLSKDPNEKRDHMVLLEFVTAAGITLGMDELYKGGTGGSMVSKGEELFTGVVPIL
VELDGDVNGHKFSVSGEGEGDATYGKLTLKFICTTGKLPVPWPTLVTTLTYGVQCFSRYPDHMKQ
HDFFKSAMPEGYIQERTIFFKDDGNYKTRAEVKFEGDTLVNRIELKGIDFKEDGNILGHKLEYNNHD
QLKRETKVLKTLSVIMGVFVCCWLPFFILNCILPFCGSGETQPFCIDSNTFDVFVWFGWANSSLNPI
IYAFNADFRKAFSTLLGCYRLCPATNNAIETVSINNNGAAMFSSHHEPRGSISKECNLVYLIPHAVG
SSEDLKKEEAAGIARPLEKLSPALSVILDYDTDVSLEKIQPITQNGQHPT*

Note: red residues highlight mutations from dLight1.3b
