# Supplementary material for: A chemogenetic approach for dopamine imaging with tunable sensitivity
Source: Nat Commun. 2024 Jul 2;15:5551. doi: 10.1038/s41467-024-49442-3 (PMC11219860; doi:10.1038/s41467-024-49442-3)
Supplement: Supplementary file 3 — Description of Additional supplementary file. [file 41467_2024_49442_MOESM3_ESM.pdf]

Description of Additional supplementary file

Supplementary Movie 1 | Imaging DA release from primary cultured dopaminergic neurons. Left, timelapse imaging of sniffer cells (green) overlaid with a static image of td-Tomato positive primary mouse dopaminergic neurons. Drugs applied to the perfusion are shown on the top left corner. Right, three dimensional $\Delta F/F0$ heatmap generated from the timelapse shown on left.
