# Supplementary material for: A chemogenetic approach for dopamine imaging with tunable sensitivity
Source: Nat Commun. 2024 Jul 2;15:5551. doi: 10.1038/s41467-024-49442-3 (PMC11219860; doi:10.1038/s41467-024-49442-3)
Supplement: Supplementary file 5 — Reporting summary [file 41467_2024_49442_MOESM5_ESM.pdf]

nature portfolio

Corresponding author(s): Tommaso Patriarchi

Last updated by author(s): May 23, 2024

Reporting Summary

Nature Portfolio wishes to improve the reproducibility of the work that we publish. This form provides structure for consistency and transparency in reporting. For further information on Nature Portfolio policies, see our Editorial Policies and the Editorial Policy Checklist.

Statistics

For all statistical analyses, confirm that the following items are present in the figure legend, table legend, main text, or Methods section.

n/a	Confirmed	
☐	☒	The exact sample size (*n*) for each experimental group/condition, given as a discrete number and unit of measurement
☐	☒	A statement on whether measurements were taken from distinct samples or whether the same sample was measured repeatedly
☐	☒	The statistical test(s) used AND whether they are one- or two-sided<br>*Only common tests should be described solely by name; describe more complex techniques in the Methods section.*
☒	☐	A description of all covariates tested
☐	☒	A description of any assumptions or corrections, such as tests of normality and adjustment for multiple comparisons
☐	☒	A full description of the statistical parameters including central tendency (e.g. means) or other basic estimates (e.g. regression coefficient) AND variation (e.g. standard deviation) or associated estimates of uncertainty (e.g. confidence intervals)
☐	☒	For null hypothesis testing, the test statistic (e.g. *F*, *t*, *r*) with confidence intervals, effect sizes, degrees of freedom and *P* value noted<br>*Give P values as exact values whenever suitable.*
☒	☐	For Bayesian analysis, information on the choice of priors and Markov chain Monte Carlo settings
☒	☐	For hierarchical and complex designs, identification of the appropriate level for tests and full reporting of outcomes
☒	☐	Estimates of effect sizes (e.g. Cohen's *d*, Pearson's *r*), indicating how they were calculated

Our web collection on statistics for biologists contains articles on many of the points above.

Software and code

Policy information about availability of computer code

Data collection

Below is the software used for data collection in this manuscript:
• Zeiss Zen, Zen Blue and Zen Black (https:/ /www.zeiss.com/microscopy/int/products/microscope-software/zen-lite.html)
• Confocal microscope (Nikon A1 HD25) (https://www.microscope.healthcare.nikon.com/en_AOM/products/confocal-microscopes/a1hd25-a1rhd25)
• Custom-built BX51WI microscope (Olympus) (https://www.olympus-lifescience.com/en/microscopes/upright/bxwi/)
• sCMOS sensor (Dhyana 400BSI V2, Tucsen Photonics) (https://www.tucsen.com/Home/Product/info/dataid/27.html)
• Doric Neuroscience Studio v6.1.2.0 (https://neuro.doriclenses.com/products/doric-neuroscience-studio)
• TDT Synapse (https://www.tdt.com/component/synapse-software/)
• MATLAB versions R2019b (https://www.mathworks.com/products/matlab.html)
• Python 2.7 (https://www.python.org)
• MicroManager2.0 (https://micro-manager.org/Download_Micro-Manager_Latest_Release)
• pClamp 10.7 (https://support.moleculardevices.com/s/article/Axon-pCLAMP-10-Electrophysiology-Data-Acquisition-Analysis-Software-Download-Page)
• EPC 10 USB Patch Clamp Amplifier (HEKA) and PATCHMASTER v2x91 (https://www.heka.com/downloads/downloads_main.html#down_patchmaster)

Data analysis

Below is the software used for data analysis in this manuscript:
• ImageJ version 1.52 (http://imagej.nih.gov/ij/download.html)
• Python 3.6 (https://www.python.org/)
• GraphPad Prism 9.0.0 (https://www.graphpad.com/scientific-software/prism/)
• MATLAB versions R2019a, R2019b, R2022a (https://www.mathworks.com/products/matlab.html)

• Anymaze (v7.2) software (Stoelting Co.) (https://www.any-maze.com/)
• DeepLabCut (version 2.1.8.2) (https://www.mackenziemathislab.org/deeplabcut)
• AxoGraph (J. Clements) (https://axograph.com/)

For manuscripts utilizing custom algorithms or software that are central to the research but not yet described in published literature, software must be made available to editors and reviewers. We strongly encourage code deposition in a community repository (e.g. GitHub). See the Nature Portfolio guidelines for submitting code & software for further information.

Data

Policy information about availability of data

All manuscripts must include a data availability statement. This statement should provide the following information, where applicable:

- Accession codes, unique identifiers, or web links for publicly available datasets
- A description of any restrictions on data availability
- For clinical datasets or third party data, please ensure that the statement adheres to our policy

DNA and protein sequences for the sensors developed in this study are available in Supplementary Note 1. DNA plasmids have been deposited at the UZH Viral Vector Facility (https://vvf.ethz.ch/). Viral vectors can be obtained either from the Patriarchi laboratory, or from the UZH Viral Vector Facility. The protein structure for dopamine-bound dopamine receptor 1 used in this work can be accessed via PDB ID 7LJD [https://doi.org/10.2210/pdb7LJD/pdb. Source data are provided with this paper. Raw data is available at https://zenodo.org/doi/10.5281/zenodo.10932251 or by emailing the corresponding author.

Research involving human participants, their data, or biological material

Policy information about studies with human participants or human data. See also policy information about sex, gender (identity/presentation), and sexual orientation and race, ethnicity and racism.

Reporting on sex and gender	*Use the terms sex (biological attribute) and gender (shaped by social and cultural circumstances) carefully in order to avoid confusing both terms. Indicate if findings apply to only one sex or gender; describe whether sex and gender were considered in study design; whether sex and/or gender was determined based on self-reporting or assigned and methods used.<br>Provide in the source data disaggregated sex and gender data, where this information has been collected, and if consent has been obtained for sharing of individual-level data; provide overall numbers in this Reporting Summary. Please state if this information has not been collected.<br>Report sex- and gender-based analyses where performed, justify reasons for lack of sex- and gender-based analysis.*
Reporting on race, ethnicity, or other socially relevant groupings	*Please specify the socially constructed or socially relevant categorization variable(s) used in your manuscript and explain why they were used. Please note that such variables should not be used as proxies for other socially constructed/relevant variables (for example, race or ethnicity should not be used as a proxy for socioeconomic status).<br>Provide clear definitions of the relevant terms used, how they were provided (by the participants/respondents, the researchers, or third parties), and the method(s) used to classify people into the different categories (e.g. self-report, census or administrative data, social media data, etc.)<br>Please provide details about how you controlled for confounding variables in your analyses.*
Population characteristics	*Describe the covariate-relevant population characteristics of the human research participants (e.g. age, genotypic information, past and current diagnosis and treatment categories). If you filled out the behavioural & social sciences study design questions and have nothing to add here, write "See above."*
Recruitment	*Describe how participants were recruited. Outline any potential self-selection bias or other biases that may be present and how these are likely to impact results.*
Ethics oversight	*Identify the organization(s) that approved the study protocol.*

Note that full information on the approval of the study protocol must also be provided in the manuscript.

Field-specific reporting

Please select the one below that is the best fit for your research. If you are not sure, read the appropriate sections before making your selection.

☒ Life sciences ☐ Behavioural & social sciences ☐ Ecological, evolutionary & environmental sciences

For a reference copy of the document with all sections, see nature.com/documents/nr-reporting-summary-flat.pdf

Life sciences study design

All studies must disclose on these points even when the disclosure is negative.

Sample size	No sample size calculation was performed. Sample sizes were based on the previous scientific literature in the field (Duffet et al, Nat Methods 2022; Patriarchi et al, Nat Methods 2020; Patriarchi et al, Science 2018).
Data exclusions	N/A

Replication	All experiments were repeated using multiple cell cultures and multiple animal subjects within each group. All replication attempts were successful.
Randomization	Group allocations used in this study were randomly assigned to animals and/or cultured cells.
Blinding	The Investigators were not blinded to allocation during experiments and outcome assessment.

Reporting for specific materials, systems and methods

We require information from authors about some types of materials, experimental systems and methods used in many studies. Here, indicate whether each material, system or method listed is relevant to your study. If you are not sure if a list item applies to your research, read the appropriate section before selecting a response.

Materials & experimental systems

n/a	Involved in the study
☐	☒ Antibodies
☐	☒ Eukaryotic cell lines
☒	☐ Palaeontology and archaeology
☐	☒ Animals and other organisms
☒	☐ Clinical data
☒	☐ Dual use research of concern
☒	☐ Plants

Methods

n/a	Involved in the study
☒	☐ ChIP-seq
☒	☐ Flow cytometry
☒	☐ MRI-based neuroimaging

Antibodies

Antibodies used

rabbit anti-GFP (1:1000; Thermo Fisher Scientific, cat#A6455);
mouse anti-TH (1:500; Immunostar cat#22941);
chicken anti-TH (1:500; Aves Labs cat#TYH0020)
mouse anti-dsRed (1:500; Takara cat#632392);
rat anti-mCherry (1:500; Molecular Probes cat#M11217);
rabbit anti-mCherry (1:500; Abcam cat#ab167453);
Alexa-488-labeled donkey anti-rabbit (1:1000, Jackson Immunoresearch; 711-546-152, RRID AB_2340619);
Cy3-labeled donkey anti-mouse (1:1000, Jackson Immunoresearch; 715-165-151);
Alexa-488-labeled donkey anti-mouse (1:1000, Jackson Immunoresearch; 715-486-150);
Alexa-546-labeled donkey anti-rat (1:1000, Jackson Immunoresearch; cat#712-546-150);
Alexa-647-labeled donkey anti-chicken (1:1000, Thermo Fisher; #A78952);
Alexa-488-labeled goat anti-rabbit (1:1000, Jackson Immunoresearch; 111-545-003)

Validation

All antibodies were validated by the commercial provider.

Eukaryotic cell lines

Policy information about cell lines and Sex and Gender in Research

Cell line source(s)	HEK293T cells (ATCC cat#3216), T-REX 293 (Thermo Scientific cat#R71007)
Authentication	The cell lines were authenticated by the vendors using Short Tandem Repeat (STR) Profiling to detect misidentified, cross-contaminated, or genetically-drifted lines.
Mycoplasma contamination	The cell lines used were mycoplasma-free.
Commonly misidentified lines (See ICLAC register)	The study did not involve commonly misidentified cell lines.

Animals and other research organisms

Policy information about studies involving animals; ARRIVE guidelines recommended for reporting animal research, and Sex and Gender in Research

Laboratory animals

Mouse pups from DATIREScre (B6.SJL-Slc6a3tm1.1(cre)Bkmn/J; Jackson Labs)31 x Ai9 (B6;129S6-Gt(ROSA)26Sor tm14(CAG-tdTomato)Hze /J; Jackson Labs)32 mice were used for preparing primary DA neuron cultures. Adult DATIREScre31, DRD1:cre (B6;129-Tg(Drd1-cre)120Mxu/Mmjax; Jackson Labs)81, or wild-type (>8 weeks old) C57/Bl6J mice of both sexes were used in this study. Mice were kept with ad libitum access to chow and water on either normal or reversed 12-h/12-h light/dark cycle for two-photon or photometry/optogenetic experiments, respectively. Behavior experiments were performed during the dark phase.

Wild animals	No wild animals were used in this study.
Reporting on sex	Mice of both sexes were used in this study.
Field-collected samples	No field collected samples were used.
Ethics oversight	All animal procedures were performed in accordance to the Animal Welfare Ordinance (TSchV 455.1) of the Swiss Federal Food Safety and Veterinary Office and were approved by the Zurich Cantonal Veterinary Office, the animal ethics committee of the Université de Montréal, or other relevant National and Institutional regulatory bodies.

Note that full information on the approval of the study protocol must also be provided in the manuscript.

Plants

Seed stocks	*Report on the source of all seed stocks or other plant material used. If applicable, state the seed stock centre and catalogue number. If plant specimens were collected from the field, describe the collection location, date and sampling procedures.*
Novel plant genotypes	*Describe the methods by which all novel plant genotypes were produced. This includes those generated by transgenic approaches, gene editing, chemical/radiation-based mutagenesis and hybridization. For transgenic lines, describe the transformation method, the number of independent lines analyzed and the generation upon which experiments were performed. For gene-edited lines, describe the editor used, the endogenous sequence targeted for editing, the targeting guide RNA sequence (if applicable) and how the editor was applied.*
Authentication	*Describe any authentication procedures for each seed stock used or novel genotype generated. Describe any experiments used to assess the effect of a mutation and, where applicable, how potential secondary effects (e.g. second site T-DNA insertions, mosiacism, off-target gene editing) were examined.*
